# Muscle‐Derived miR‐200a‐3p Through Light‐Intensity Exercise May Contribute to Improve Memory Dysfunction in Type 2 Diabetic Mice

**DOI:** 10.1096/fj.202500336R

**Published:** 2025-04-10

**Authors:** Takeru Shima, Hayate Onishi, Chiho Terashima

**Affiliations:** ^1^ Department of Health and Physical Education, Cooperative Faculty of Education Gunma University Maebashi Gunma Japan; ^2^ Graduate School of Medicine Gunma University Maebashi Gunma Japan

**Keywords:** exercise, exosome, gastrocnemius muscle, memory function, miR‐200a‐3p, type 2 diabetes

## Abstract

Memory dysfunction associated with type 2 diabetes mellitus (T2DM) poses a threat to well‐being. Engaging in light‐intensity exercise has favorable effects on hippocampal function and molecular profiles, including *Mct2* mRNA and miR‐200a‐3p. Here, we investigated the involvement of exosomal miR‐200a‐3p secretion from gastrocnemius muscles in T2DM mice undergoing light‐intensity exercise intervention, focusing on its potential to ameliorate memory dysfunction. We initially assessed the effects of light‐intensity exercise over a 4‐week period on memory function, the secretion of gastrocnemius muscle‐derived exosomal miR‐200a‐3p, and hippocampal mRNA. Subsequently, the impact of a daily intraperitoneal injection of the mmu‐miR‐200a‐3p mimic over a 4‐week duration on hippocampal dysregulation in ob/ob mice was investigated. The light‐intensity exercise intervention improved gastrocnemius muscle‐derived and plasma exosomal miR‐200a‐3p levels in ob/ob mice, concomitant with improved memory dysfunction. Intriguingly, the daily intraperitoneal injection of the mmu‐miR‐200a‐3p mimic also improved memory function in ob/ob mice. Notably, both the exercise intervention and miR‐200a‐3p mimic treatment induced downregulation in hippocampal *Keap1* and upregulation in *Hsp90aa1* and *Mct2* mRNA in ob/ob mice. These results imply that the augmentation of peripherally derived miR‐200a‐3p contributes to ameliorating memory dysfunction in T2DM mice undergoing light‐intensity exercise, with a possible contribution from gastrocnemius muscle‐derived exosomal miR‐200a‐3p to these exercise effects.

## Graphical abstract

The findings suggest that light‐intensity exercise restores gastrocnemius muscle‐derived exosomal miR‐200a‐3p levels, improving spatial memory and hippocampal *Mct2* mRNA levels in ob/ob mice, a type 2 diabetes model. Additionally, miR‐200a‐3p mimic injections alleviated memory dysfunction and replicated exercise‐induced hippocampal RNA changes. Thus, peripherally derived miR‐200a‐3p may aid memory improvement in T2DM mice, with gastrocnemius‐derived exosomal miR‐200a‐3p potentially contributing to these effects.

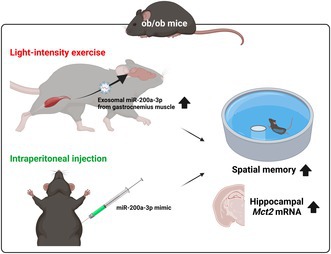

## 1 Introduction

In light of the favorable impact that physical exercise exerts on both peripheral organs and the brain, it has emerged as a widely adopted therapeutic strategy for addressing metabolic syndrome and mental health disorders [1]. Within the context of type 2 diabetes (T2DM), exercise therapy primarily focuses on mitigating insulin resistance and enhancing glycemic control [2, 3]. T2DM triggers various complications not only in peripheral organs but also in the hippocampus, leading to memory dysfunction [4, 5, 6]. It is anticipated that exercise can serve as a therapeutic intervention for memory dysfunction in T2DM [7]. Therefore, elucidating the mechanisms underlying the effects of exercise on the hippocampus in T2DM holds the potential to develop a therapeutic approach targeting the hippocampus in T2DM, a crucial endeavor for the preservation of human well‐being.

The downregulation of lactate transport through monocarboxylate transporter 2 (MCT2) into neurons is assumed to be a potential etiological mechanism underlying T2DM‐induced memory dysfunction [8, 9, 10, 11]. Lactate is a crucial energy substrate for neurons, derived from blood circulation and astrocytes through glycolysis and glycogenolysis pathways [12, 13, 14, 15]. Within astrocytes, glycogen functions as a principal reservoir for lactate. The release of glycogen‐derived lactate from astrocytes into the extracellular fluid is facilitated by transporters MCT1 and MCT4, subsequently taken up by neurons through MCT2 [15]. Neurons utilize lactate both as an energy substrate for their activity and as a neuromodulator, contributing to neuronal plasticity [16, 17, 18]. Previous studies have demonstrated that hindering lactate supply and downregulating MCT2 expression and function within the hippocampus result in impairments in learning and memory [16, 19, 20, 21, 22]. Significantly, rodents with T2DM exhibit reduced expression levels of MCT2 in their hippocampi without notable differences in MCT1 or MCT4 expressions compared to control rodents [8, 9, 10, 11]. Research has shown that a light‐intensity exercise intervention improves hippocampal memory dysfunction and enhances MCT2 expression in rodent models of T2DM [10, 11]. Consequently, the compromised transport of lactate through MCT2 likely plays a pivotal role in the hippocampal complications associated with memory dysfunction in T2DM, shedding light on the potential therapeutic effects of exercise.

Several studies have proposed that the release of exosomes constitutes a fundamental mechanism underpinning the effects of exercise. These exosomes, characterized as small extracellular vesicles within the range of 40 to 160 nm, are released by diverse organs and encapsulate microRNAs (miRNAs). These miRNAs, short non‐coding RNAs measuring approximately 21–25 nucleotides, engage in binding with complementary segments of messenger RNAs (mRNAs), thereby initiating mRNA degradation or hindering translation [23]. Notably, emerging evidence suggests that exercise‐induced exosomal miRNAs, originating from muscles, contribute to enhanced biological and physiological functions in peripheral organs and the brain [24, 25, 26, 27]. However, it remains unclear whether exosomal miRNAs released during light‐intensity exercise are associated with the improvement of hippocampal function in the context of T2DM.

A prior investigation has documented that light‐intensity exercise intervention increases miR‐200a‐3p levels, accompanied by the upregulation of *Mct2* mRNA levels in the hippocampus [11]. Although the changing profile of miR‐200a‐3p in the hippocampus of T2DM patients is not well understood, the downregulation of miR‐200a‐3p is associated with diabetic complications such as retinopathy and cardiomyopathy [28, 29]. Reduced miR‐200a‐3p levels have been observed in the hippocampus of mice with Alzheimer's disease (AD) and aging, with evidence suggesting that miR‐200a‐3p plays a role in inhibiting apoptosis and amyloid‐β production [30]. Furthermore, miR‐200a‐3p is notable for its role in cell growth and its inhibitory effect on mRNA levels in Kelch ECH‐associated protein 1 (*Keap1*) and phosphatase and tensin homolog (*Pten*) [31, 32]. KEAP1 functions as a negative regulator for nuclear factor erythroid 2 p45‐related factor‐2 (NRF2) [33] and heat shock protein 90 (HSP90) [34]. In the context of T2DM, there is observed overexpression of KEAP1 in various organs, including the hippocampus, which is hypothesized to be a potential contributor to complications associated with T2DM [35, 36, 37]. Notably, it has been reported that high‐intensity interval exercise intervention improves the overexpression of KEAP1 in the T2DM hippocampus [37]. Furthermore, PTEN is implicated in T2DM pathogenesis [38] and has an impact on the expressions of brain‐derived neurotrophic factor (BDNF) [39]. Previous studies have proposed potential associations between profiles of HSPs and the expressions of MCTs [40, 41], while BDNF is known to modulate MCT2 expressions [42, 43]. Thus, the restoration of miR‐200a‐3p levels in the hippocampus may contribute to the amelioration of memory dysfunction and the enhancement of hippocampal MCT2 expressions in T2DM through the modulation of mRNA levels in *Keap1* and *Pten*. Furthermore, a previous study has indicated that 4 weeks of light‐intensity exercise did not significantly alter circulating miR‐200a‐3p levels in healthy mice [44]; however, the impact of such exercise intervention on circulating miR‐200a‐3p levels in T2DM mice remains uncertain.

Here, we initially evaluated the impact of light‐intensity exercise intervention on the release of exosomal miR‐200a‐3p from gastrocnemius muscles in T2DM mice, simultaneously addressing improvement in memory dysfunction and alterations in hippocampal mRNA expressions. Subsequently, we conducted a detailed investigation into the effects of daily intraperitoneal injections of mmu‐miR‐200a‐3p mimic on both hippocampal memory function and mRNA expressions in T2DM mice.

## 2 Materials and Methods

### 2.1 Animals

Male C57BL/6 mice and ob/ob mice (a T2DM mouse model) at 8 weeks of age were obtained from SLC Inc. (Japan) and housed in a temperature‐controlled room (21–23°C) with a 12‐h light/dark cycle (lights on from 7 AM to 7 PM). They were provided ad libitum access to a standard pellet diet (Rodent Diet CE‐2; CLEA Japan Inc., Japan) and water. The experimental procedures were pre‐approved by the Gunma University Animal Care and Experimentation Committee (approval No. 22‐012) and conducted according to the Japanese Act on the Welfare and Management of Animals and the Guidelines for the Proper Conduct of Animal Experiments issued by the Science Council of Japan and ARRIVE guidelines 2.0.

### 2.2 Exercise Training

Following a week of acclimatization, both C57BL/6 mice and ob/ob mice at 9 weeks of age were divided into exercise and non‐exercise (sedentary) groups, ensuring a match in body weight between the groups. The groups were as follows: sedentary C57BL/6 (*n* = 10), exercised C57BL/6 (*n* = 9), sedentary ob/ob (*n* = 9), and exercised ob/ob (*n* = 9). Mice in the exercise group underwent a running habituation phase on a forced exercise wheel bed for 30 min/day, 5 days/week (a total of five sessions over 6 days) at speeds ranging from 3.0 to 7.0 m/min for C57BL/6 mice and from 3.0 to 5.0 m/min for ob/ob mice, spanning 1 week. Subsequently, they engaged in light‐intensity exercise sessions (C57BL/6 mice, 7.0 m/min; ob/ob mice, 5.0 m/min) on the same equipment for 30 min/day, 5 days/week over 3 weeks [11, 45, 46]. The running speeds were determined based on the ventilatory threshold to ensure equivalent relative exercise intensity across strains [46]. All sessions were conducted during the light period (from 7 AM to 9 AM). Memory performance tests were conducted for both exercise and sedentary groups for 5 days during the final week of the exercise regimen.

### 2.3 Intraperitoneal Injection of mmu‐miR‐200a‐3p Mimic

After 1 week of acclimatization, the mice at 9 weeks of age were divided into miR‐200a‐3p mimic‐treated and miRNA mimic negative control‐treated groups, ensuring that body weights were matched between the groups. Mice in the miR‐200a‐3p mimic‐treated group and the miRNA mimic negative control‐treated group received daily intraperitoneal injections of mmu‐miR‐200a‐3p mimic (Ajinomoto Bio‐Pharma, Japan) and miRNA mimic negative control (mimic NC; SMC‐2003, Bioneer, Korea) for 4 weeks, respectively. A 20 nmol/L solution of each drug was prepared by diluting it in 0.9% saline with 0.02% TE buffer. Mice received an injection of 10 μL/g body weight of either the mmu‐miR‐200a‐3p mimic or the mimic NC solution during the light period (0.2 nmol/kg body weight, once a day from 8 AM to 9 AM). The designated groups were as follows: C57BL/6 mimic NC (*n* = 8), C57BL/6 miR‐200a‐3p mimic (*n* = 8), ob/ob mimic NC (*n* = 8), and ob/ob miR‐200a‐3p mimic (*n* = 8). These mice did not undergo exercise training. Memory performance tests were conducted for all mice over 5 days during the final week of the treatment.

### 2.4 Memory Performance Test

The Morris water maze test was conducted in a circular pool (100 cm in diameter and 30 cm in depth) with an invisible platform (10 cm in diameter) positioned in the center of one quadrant. The experimental room had several extra‐maze cues. All four start points were utilized during the learning sessions in different sequences. Mice were given 60 s to explore and locate the platform. In cases where mice failed to find the platform within 60 s, they were manually guided to it. Upon reaching the platform, the mice remained there for 10 s. Throughout the learning sessions, escape latency (sec), swim length (cm), and speed (cm/s) were recorded using a video tracking system (O'hara & Co. Ltd., Japan). One day after the final learning session, the platform was removed from the pool, and the mice underwent a probe trial for 60 s to search for it within the pool. The time spent in the quadrants where the platform had been located during the learning sessions was measured using the same tracking system.

### 2.5 Tissue Preparation

Two days after the probe trial and the end of exercise or miRNA mimic treatments, mice were anesthetized using isoflurane (Dainippon Sumitomo Pharma Co., Japan), and the blood samples were obtained from cardiac puncture. Blood glucose and HbA_1C_ levels were measured by a glucometer (FreeStyle Libre; Abbot, Japan) and an A1CNow + kit (Finggal Link Co. Ltd., Japan), respectively. The fat around the kidneys of each animal was also taken and measured. Subsequently, the hippocampus was collected and preserved in RNAlater Stabilization Solution (Invitrogen, USA). The hippocampal tissues were stored at −20°C for subsequent biochemical analysis. Furthermore, gastrocnemius muscle and plasma were collected and used to extract exosomes.

### 2.6 Extraction of RNAs and Exosomal miRNAs

Total RNAs and miRNAs were extracted from the hippocampal tissue using the RNeasy Mini Kit and the miRNeasy Micro Kit (Qiagen Inc., USA), respectively. Gastrocnemius muscle tissues obtained from mice were promptly incubated in DMEM (Gibco‐Thermo Fisher Scientific Inc., USA) supplemented with 10% exosome‐depleted FBS (EXO‐FBSHI‐50A‐1; SBI LLC., USA) and 1% Pen‐Strep‐Glutamine (Gibco‐Thermo Fisher Scientific Inc., USA) for 24 h in a humidified incubator at 37°C and 5% CO_2_. The medium was then collected and filtered through 22‐μm filters. Gastrocnemius muscle‐derived exosomal miRNAs in the medium were extracted using the exoRNeasy Midi Kit (Qiagen Inc., USA). Exosomal miRNAs and total miRNAs in plasma samples were also extracted using the exoRNeasy Midi Kit and miRNeasy Serum/Plasma Advanced Kit (Qiagen Inc., USA), respectively. As per the protocol provided by Qiagen Inc. (USA), 150 μL of plasma in each mouse was utilized to extract exosomal miRNAs or total miRNAs.

### 2.7 Real‐Time PCR

After extracting RNAs from the hippocampus, DNase I treatment was applied, and RNA quantification was carried out using the Qubit 4.0 (Invitrogen, USA). For the detection of mRNA levels in the hippocampus, 1000 ng of RNA underwent reverse transcription to cDNA using the GeneAce cDNA Synthesis Kit (Nippon Gene, Japan). Subsequently, the mRNA levels of target genes were assessed using 5.0 ng of cDNA, primers for each target gene, and the PowerTrack SYBR Green Master Mix in the StepOne Plus Real‐Time PCR 96‐well system (Thermo Fisher Scientific Inc., USA). The primer sequences (forward and reverse) used in the current study are provided in Table S1. The relative levels of each mRNA were calculated utilizing the ΔΔ*C*_T_ method and normalized by β‐actin mRNA levels.

To detect exosomal miRNA levels in the gastrocnemius muscle and plasma, as well as miRNA levels in the hippocampus and plasma, 10 ng of miRNA underwent reverse transcription to cDNA using the Taqman MicroRNA Reverse Transcription Kit and the Taqman MicroRNA Assay (miR‐200a‐3p: 000502, and U6 snRNA: 001973; Thermo Fisher Scientific Inc., USA). Subsequently, miR‐200a‐3p and U6 levels were quantified using 0.67 ng of cDNA, the Taqman MicroRNA Assay, and the Taqman Fast Advanced Master Mix in the StepOne Plus (Thermo Fisher Scientific Inc., USA). The relative levels of miR‐200a‐3p were calculated by the ΔΔ*C*_T_ method and normalized by U6 snRNA levels.

### 2.8 Statistical Analysis

The data are presented as mean with 95% confidence intervals and were analyzed using Prism version 10.1.1 (MDF, Japan). Group comparisons were conducted utilizing repeated three‐way ANOVA (factor 1: day in the Morris water maze test; factor 2: diabetes [C57BL/6 mice vs. ob/ob mice]; factor 3: exercise [sedentary vs. exercised] or miR‐200a‐3p [mimic NC vs. miR‐200a‐3p mimic]) or two‐way ANOVA (factor 1: diabetes [C57BL/6 mice vs. ob/ob mice]; factor 2: exercise [sedentary vs. exercised] or miR‐200a‐3p [mimic NC vs. miR‐200a‐3p mimic]) with Tukey's post hoc tests. We used repeated three‐way ANOVA solely for analyzing the results of escape latency, swim length, and speed during the learning sessions of the Morris water maze test. Correlations were analyzed by Pearson correlation. Statistical significance was set at *p* < 0.05.

## 3 Results

### 3.1 Effects of Light‐Intensity Exercise on Physiological and Biochemical Variables

Both sedentary and exercised ob/ob mice exhibited significantly higher body weight and fat‐to‐body weight ratio compared to both sedentary and exercised C57BL/6 mice (Table S2; *body weight*, effects of diabetes: *F*_(1, 33)_ = 1353.6, *p* < 0.0001, effects of exercise: *F*_(1, 33)_ = 0.99, *p* = 0.3280, interaction: *F*_(1, 33)_ = 0.78, *p* = 0.3831; *fat‐to‐body weight ratio*, effects of diabetes: *F*_(1, 33)_ = 2046.9, *p* < 0.0001, effects of exercise: *F*_(1, 33)_ = 3.05, *p* = 0.0899, interaction: *F*_(1, 33)_ = 1.25, *p* = 0.2711). Sedentary ob/ob mice exclusively demonstrated elevated blood glucose levels in comparison to both sedentary and exercised C57BL/6 mice, whereas exercised ob/ob mice did not exhibit this distinction (effects of diabetes: *F*_(1, 33)_ = 15.33, *p* = 0.0004, effects of exercise: *F*_(1, 33)_ = 2.73, *p* = 0.1081, interaction: *F*_(1, 33)_ = 0.88, *p* = 0.3548). Although HbA_1C_ levels were markedly higher in both groups of ob/ob mice compared to sedentary and exercised C57BL/6 mice, those in exercised ob/ob mice were significantly lower than those in sedentary ob/ob mice (effects of diabetes: *F*_(1, 33)_ = 344.4, *p* < 0.0001, effects of exercise: *F*_(1, 33)_ = 12.55, *p* = 0.0012, interaction: *F*_(1, 33)_ = 11.50, *p* = 0.0018).

### 3.2 Effects of Light‐Intensity Exercise on Memory Function

**FIGURE 1:**
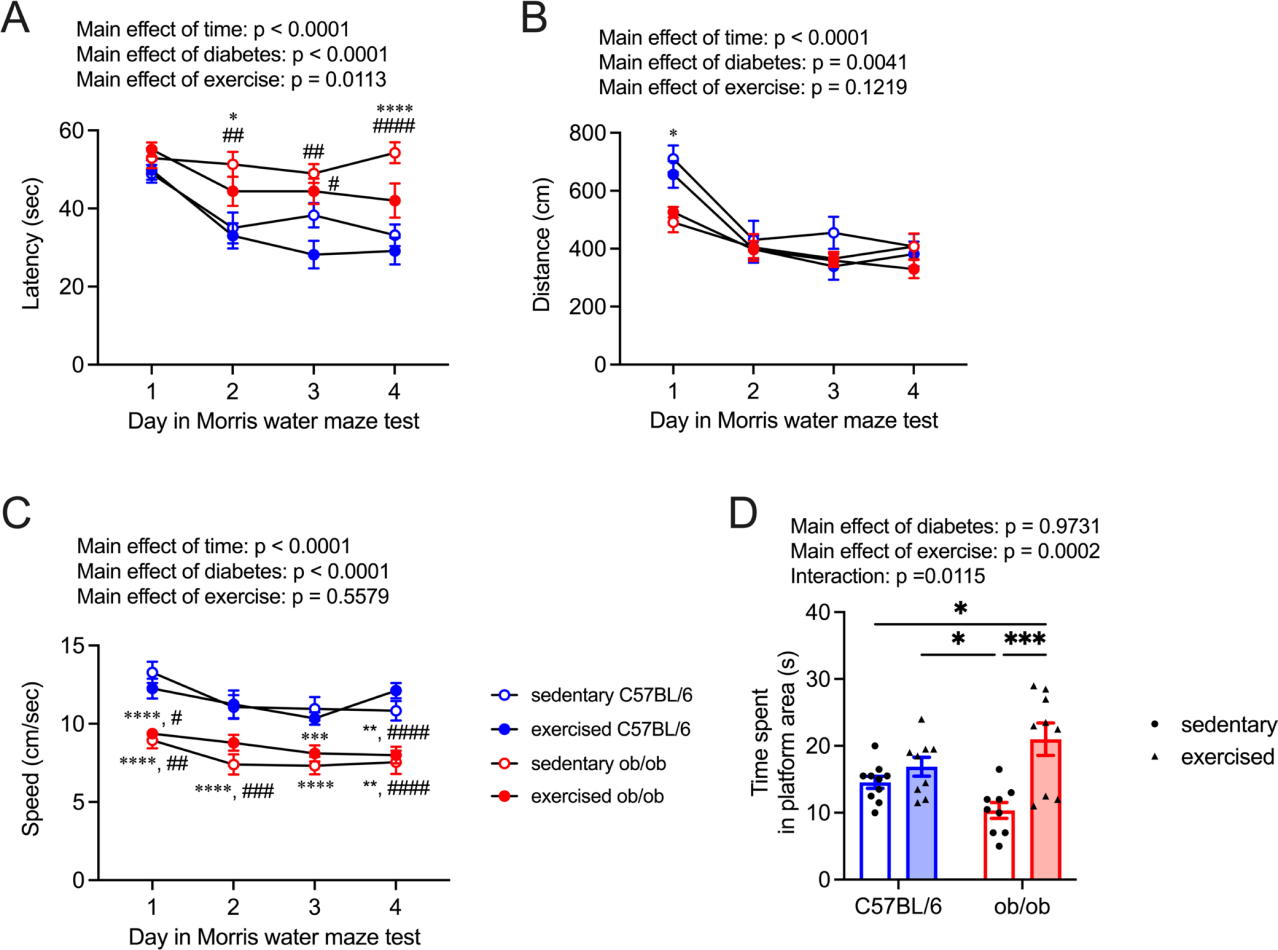
Effect of light‐intensity exercise on learning and memory function. Escape latency (A), swim length (B), and speed (C) during the learning session in mice (mean with 95% confidence intervals). Blue circles: C57BL/6 mice, red circles: Ob/ob mice. *p  <  0.05, **p < 0.01, ***p  <  0.001, ****p  <  0.0001 versus sedentary C57BL/6, ^#^ *p* < 0.05, ^##^ *p* < 0.01, ^###^ *p*  <  0.001, ^####^ *p*  <  0.0001 versus exercised C57BL/6. (D) Effect of exercise on the probe trial, showing the time spent in the platform area. Blue bars: C57BL/6, red bars: Ob/ob, circles: Sedentary groups, and triangles: Exercised groups. *p < 0.05, ***p < 0.001. Data are expressed as a mean with 95% confidence intervals, *n* = 10 mice for sedentary C57BL/6, and *n* = 9 mice for exercised C57BL/6, sedentary ob/ob, and exercised ob/ob.

Sedentary ob/ob mice exhibited significantly prolonged escape latency compared to both sedentary and exercised C57BL/6 mice, whereas exercised ob/ob mice did not exhibit this difference on 2nd and 4th day of the learning session (Figure 1A; effects of day: *F*_(3, 432)_ = 15.57, effects of diabetes: *F*_(1, 144)_ = 62.86, effects of exercise: *F*_(1, 144)_ = 6.59, interaction [day × diabetes]: *F*_(3, 432)_ = 3.68, *p* = 0.0122, interaction [day × exercise]: *F*_(3, 432)_ = 2.03, *p* = 0.1088, interaction [diabetes × exercise]: *F*_(1, 144)_ = 0.11, *p* = 0.7370, interaction [day × diabetes × exercise]: *F*_(3, 432)_ = 1.09, *p* = 0.3524). Swim distance and swimming speed were influenced by time and T2DM but not by exercise (Figure 1B,C; *swim distance*, effects of day: *F*_(3, 432)_ = 22.76, effects of diabetes: *F*_(1, 144)_ = 8.51, effects of exercise: *F*_(1, 144)_ = 2.42, interaction [day × diabetes]: *F*_(3, 432)_ = 3.17, *p* = 0.0244, interaction [day × exercise]: *F*_(3, 432)_ = 0.46, *p* = 0.7132, interaction [diabetes × exercise]: *F*_(1, 144)_ = 1.21, *p* = 0.2734, interaction [day × diabetes × exercise]: *F*_(3, 432)_ = 0.84, *p* = 0.4704; *swimming speed*, effects of day: *F*_(3, 432)_ = 9.28, effects of diabetes: *F*_(1, 144)_ = 116.9, effects of exercise: *F*_(1, 144)_ = 0.35, interaction [day × diabetes]: *F*_(3, 432)_ = 1.16, *p* = 0.3239, interaction [day × exercise]: *F*_(3, 432)_ = 1.32, *p* = 0.2685, interaction [diabetes × exercise]: *F*_(1, 144)_ = 2.55, *p* = 0.1127, interaction [day × diabetes × exercise]: *F*_(3, 432)_ = 1.87, *p* = 0.1342). During the probe test, the time spent in the platform area by exercised ob/ob mice was significantly greater than that observed in sedentary C57BL/6 mice and ob/ob mice, respectively (Figure 1D; effects of diabetes: *F*_(1, 33)_ = 0.001, effects of exercise: *F*_(1, 33)_ = 17.49, interaction: *F*_(1, 33)_ = 7.17). In contrast, sedentary ob/ob mice exhibited significantly shorter times spent in the platform area compared to exercised C57BL/6 mice (Figure 1D).

### 3.3 Effects of Light‐Intensity Exercise on mRNA Levels Related to Lactate Transport in the Hippocampus

**FIGURE 2:**
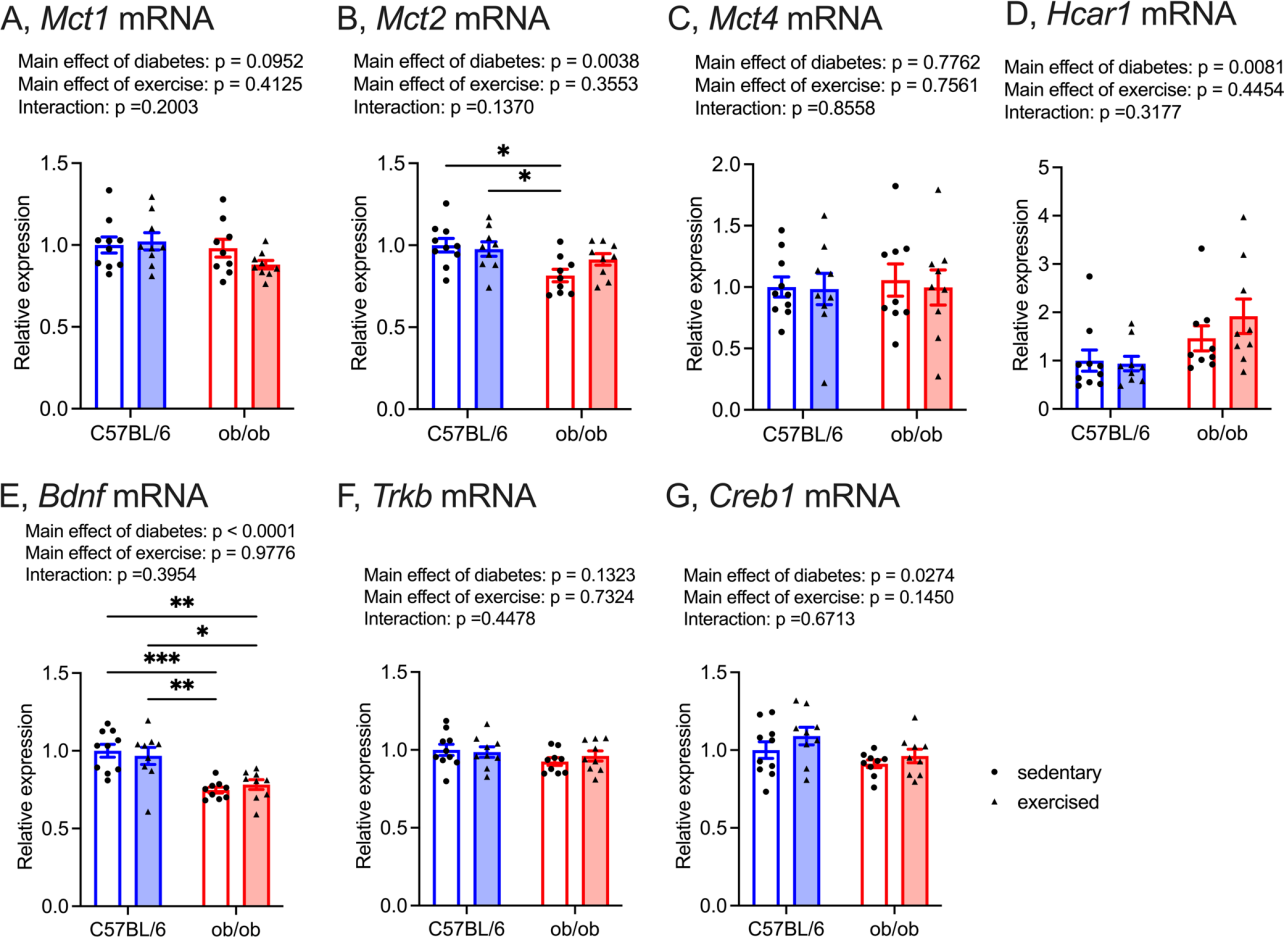
Effect of light‐intensity exercise on mRNA levels of *Mct1* (A), *Mct2* (B), *Mct4* (C), *Hcar1* (D), *Bdnf* (E), *Trkb* (F), *Creb1* (G) in the hippocampus. The sedentary C57BL/6 group was normalized as 1.0. Data are expressed as a mean with 95% confidence intervals, *n* = 10 mice for sedentary C57BL/6, and *n* = 9 mice for exercised C57BL/6, sedentary ob/ob, and exercised ob/ob. *p < 0.05, **p < 0.01, ***p < 0.001.

The sedentary ob/ob mice exhibited notably reduced hippocampal *Mct2* mRNA levels compared to both sedentary and exercised C57BL/6 mice; however, this difference was not observed in exercised ob/ob mice (Figure 2B; effects of diabetes: *F*_(1, 33)_ = 9.69, effects of exercise: *F*_(1, 33)_ = 0.88, interaction: *F*_(1, 33)_ = 2.32). The mRNA levels of *Mct1* and *Mct4* in the hippocampus remained unaltered regardless of T2DM or exercise (Figure 2A,C; *Mct1*, effects of diabetes: *F*_(1, 33)_ = 2.95, effects of exercise: *F*_(1, 33)_ = 0.69, interaction: *F*_(1, 33)_ = 1.71; *Mct4*, effects of diabetes: *F*_(1, 33)_ = 0.08, effects of exercise: *F*_(1, 33)_ = 0.10, interaction: *F*_(1, 33)_ = 0.03). In contrast, hippocampal *Hcar1* mRNA levels were significantly elevated in mice with T2DM compared to control mice (Figure 2D; effects of diabetes: *F*_(1, 33)_ = 7.93, effects of exercise: *F*_(1, 33)_ = 0.60, interaction: *F*_(1, 33)_ = 1.03). Both sedentary and exercised ob/ob mice showed significantly diminished *Bdnf* mRNA levels in the hippocampus compared to both sedentary and exercised C57BL/6 mice (Figure 2E; effects of diabetes: *F*_(1, 33)_ = 31.11, effects of exercise: *F*_(1, 33)_ = 0.001, interaction: *F*_(1, 33)_ = 0.74). Although hippocampal tropomyosin‐related kinase B (*Trkb*) mRNA levels remained unaffected by T2DM or exercise (Figure 2F; effects of diabetes: *F*_(1, 33)_ = 2.38, effects of exercise: *F*_(1, 33)_ = 0.12, interaction: *F*_(1, 33)_ = 0.59), mRNA levels of cAMP response element binding protein (*Creb1*) in the hippocampus were lower in T2DM mice compared to control mice (Figure 2G; effects of diabetes: *F*_(1, 33)_ = 5.33, effects of exercise: *F*_(1, 33)_ = 2.23, interaction: *F*_(1, 33)_ = 0.18).

### 3.4 Effects of Light‐Intensity Exercise on Exosomal and Hippocampal miR‐200a‐3p and Its‐Related mRNA Levels in the Hippocampus

**FIGURE 3:**
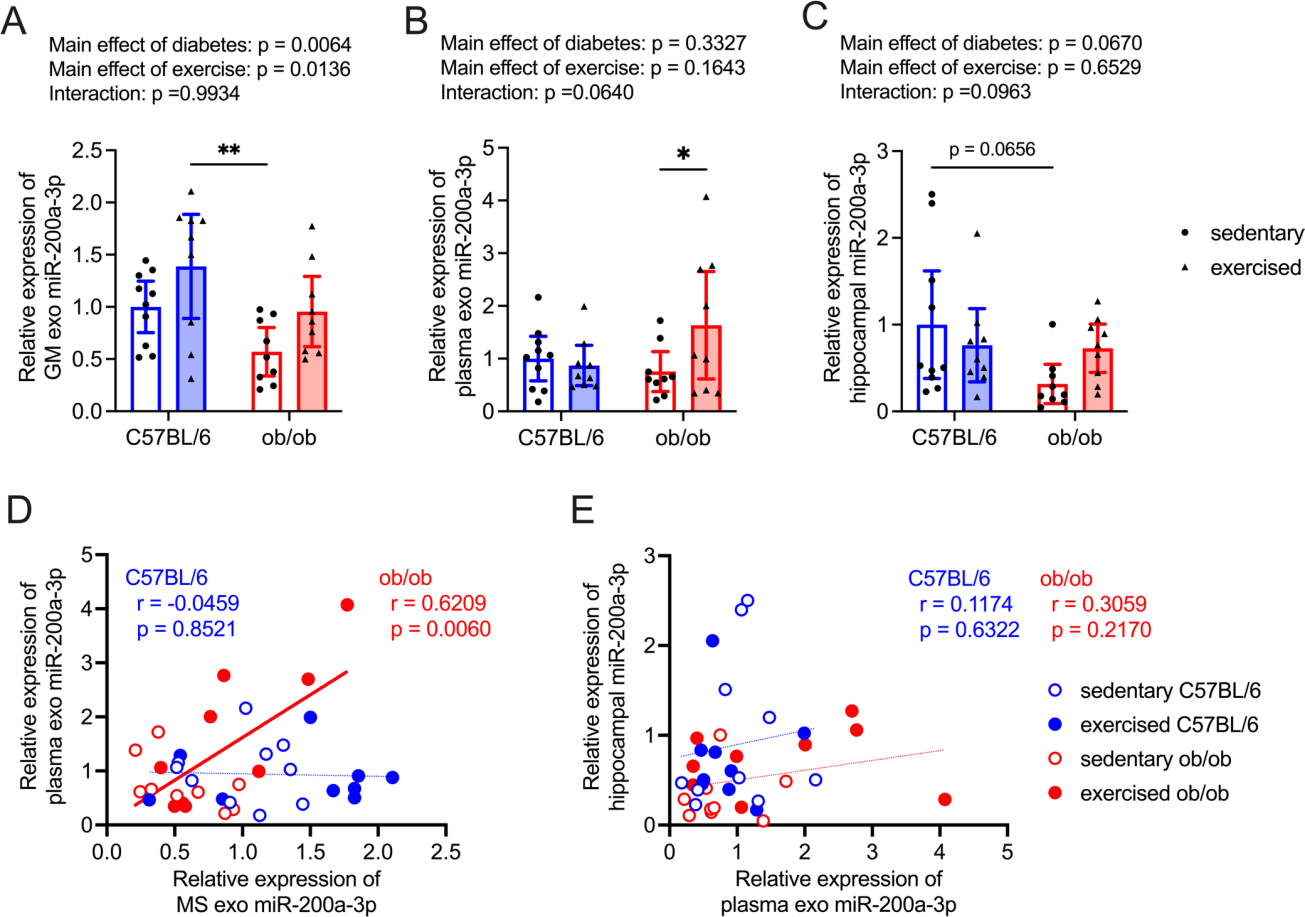
Effect of light intensity exercise on the release of exosomal miR‐200a‐3p. Effect of light‐intensity exercise on gastrocnemius muscle‐derived exosomal miR‐200a‐3p (A), exosomal miR‐200a‐3p in plasma (B), and miR‐200a‐3p in hippocampus. (C) The sedentary C57BL/6 group was normalized as 1.0. Data are expressed as mean with 95% confidence intervals, *n* = 10 mice for sedentary C57BL/6, and *n* = 9 mice for exercised C57BL/6, sedentary ob/ob, and exercised ob/ob. *p < 0.05, **p < 0.01. (D) The correlations between gastrocnemius muscle‐derived exosomal miR‐200a‐3p and exosomal miR‐200a‐3p in plasma. The red line in the scatter diagram indicates a significant correlation in ob/ob mice. (E) The correlations between exosomal miR‐200a‐3p in plasma and hippocampal miR‐200a‐3p levels.

Although the presence of T2DM led to a downregulation of exosomal miR‐200a‐3p levels derived from the gastrocnemius muscle, these levels were increased following a light‐intensity exercise intervention (Figure 3A; effects of diabetes: *F*_(1, 33)_ = 8.47, effects of exercise: *F*_(1, 33)_ = 6.80, interaction: *F*_(1, 33)_ = 0.0001). Specifically, sedentary ob/ob mice exhibited significantly lower gastrocnemius muscle‐derived exosomal miR‐200a‐3p levels compared to exercised C57BL/6 mice (Figure 3A). Light‐intensity exercise significantly enhanced exosomal miR‐200a‐3p levels in the plasma of ob/ob mice (Figure 3B; effects of diabetes: *F*_(1, 33)_ = 0.97, effects of exercise: *F*_(1, 33)_ = 2.02, interaction: *F*_(1, 33)_ = 3.67). Hippocampal miR‐200a‐3p levels showed a tendency to decrease in sedentary ob/ob mice compared to sedentary C57BL/6 mice, but this tendency was not observed in exercised ob/ob mice (Figure 3C; effects of diabetes: *F*_(1, 33)_ = 3.59, effects of exercise: *F*_(1, 33)_ = 0.21, interaction: *F*_(1, 33)_ = 2.93). A positive association between gastrocnemius muscle‐derived exosomal miR‐200a‐3p levels and plasma exosomal miR‐200a‐3p levels was evident in ob/ob mice but not in C57BL/6 mice (Figure 3D). Additionally, a weak, albeit nonsignificant, correlation was observed between exosomal miR‐200a‐3p levels in plasma and hippocampal miR‐200a‐3p levels in ob/ob mice but not in C57BL/6 mice (Figure 3E).

**FIGURE 4:**
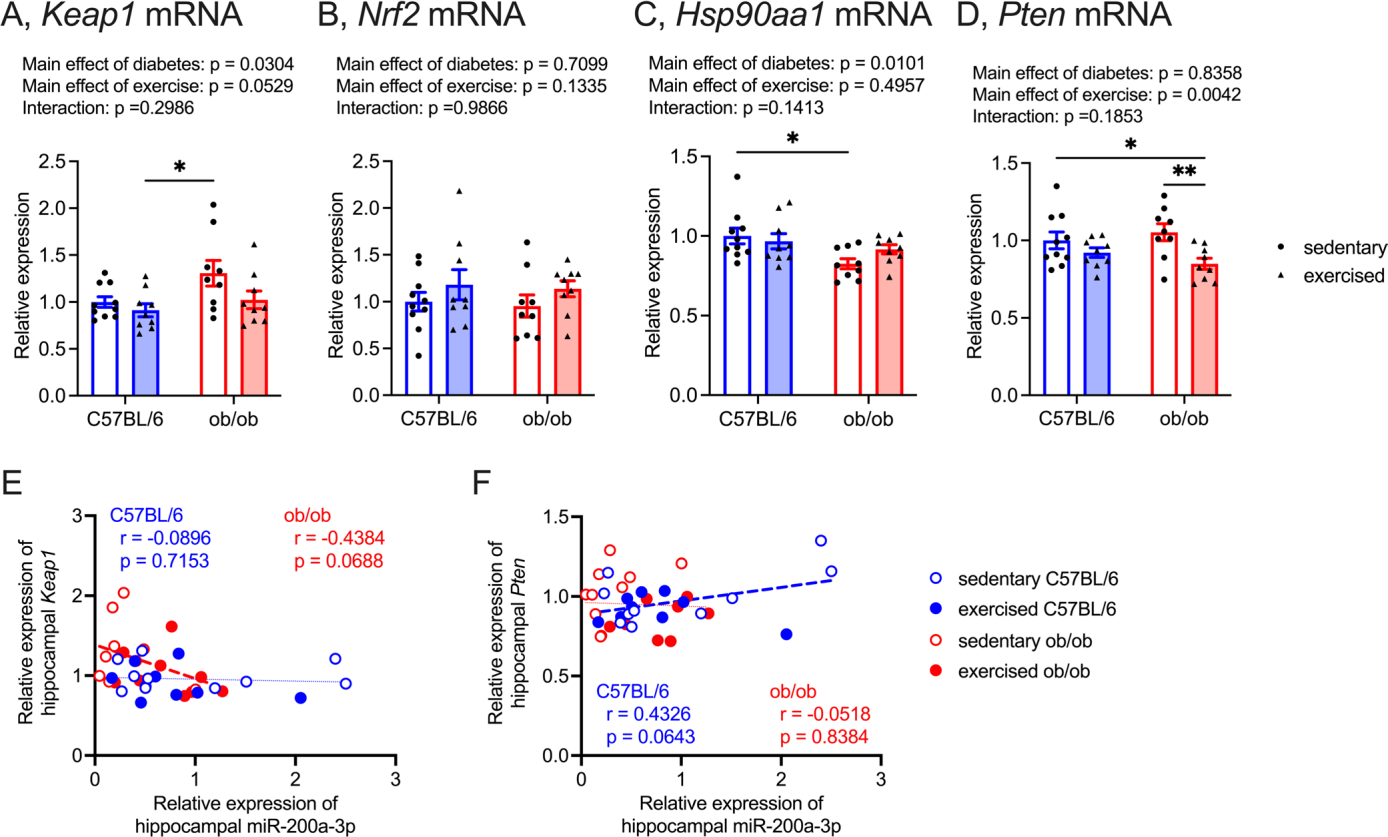
Effect of light‐intensity exercise on mRNA levels of *Keap1* (A), *Nrf2* (B), *Hsp90aa1* (C), and *Pten* (D) in the hippocampus. The sedentary C57BL/6 group was normalized as 1.0. Data are expressed as a mean with 95% confidence intervals, *n* = 10 mice for sedentary C57BL/6, and *n* = 9 mice for exercised C57BL/6, sedentary ob/ob, and exercised ob/ob. *p < 0.05, **p < 0.01. (E) The correlations between miR‐200a‐3p levels and *Keap1* mRNA levels in the hippocampus. The red dashed line in the scatter diagram indicates a trend of correlation in ob/ob mice. (F) The correlations between miR‐200a‐3p levels and *Keap1* mRNA levels in the hippocampus. The blue dashed line in the scatter diagram indicates a trend of correlation in C57BL/6 mice.

Hippocampal *Keap1* mRNA levels were significantly higher in T2DM mice than in control mice (Figure 4A). They exhibited a tendency to decrease with light‐intensity exercise intervention (Figure 4A; effects of diabetes: *F*_(1, 33)_ = 5.12, effects of exercise: *F*_(1, 33)_ = 4.03, interaction: *F*_(1, 33)_ = 1.12). Sedentary ob/ob mice exhibited significantly higher hippocampal *Keap1* mRNA levels than exercised C57BL/6 mice (Figure 4A). A trend of negative correlation was observed between the levels of miR‐200a‐3p and *Keap1* mRNA levels in the hippocampus of ob/ob mice (Figure 4E). Hippocampal *Nrf2* mRNA levels remained unaffected by T2DM or light‐intensity exercise intervention (Figure 4B; effects of diabetes: *F*_(1, 33)_ = 0.14, effects of exercise: *F*_(1, 33)_ = 2.37, interaction: *F*_(1, 33)_ = 0.0003). However, only sedentary ob/ob mice exhibited significantly lowered *Hsp90aa1* mRNA levels in the hippocampus compared to sedentary C57BL/6 mice (Figure 4C; effects of diabetes: *F*_(1, 33)_ = 7.45, effects of exercise: *F*_(1, 33)_ = 0.48, interaction: *F*_(1, 33)_ = 2.27). Although hippocampal *Pten* mRNA levels were significantly downregulated with light‐intensity exercise intervention (Figure 4D; effects of diabetes: *F*_(1, 33)_ = 0.04, effects of exercise: *F*_(1, 33)_ = 9.46, interaction: *F*_(1, 33)_ = 1.83), no correlation was found between these levels and hippocampal miR‐200a‐3p levels in ob/ob mice (Figure 4F).

### 3.5 Effects of Intraperitoneal Injection of miR‐200a‐3p Mimic on Physiological and Biochemical Variables

Both miRNA mimic NC‐treated and miR‐200a‐3p mimic‐treated ob/ob mice exhibited increased body weight, fat‐to‐body weight ratio, elevated blood glucose levels, and higher HbA_1C_ levels compared to miRNA mimic NC‐treated and miR‐200a‐3p mimic‐treated C57BL/6 mice (Table S3). No difference in these parameters was observed between miRNA mimic NC‐treated and miR‐200a‐3p mimic‐treated ob/ob mice (*body weight*, effects of diabetes: *F*_(1, 28)_ = 578.2, *p* < 0.0001, effects of miR‐200a‐3p: *F*_(1, 28)_ = 0.25, *p* = 0.6231, interaction: *F*_(1, 28)_ = 0.002, *p* = 0.9698; *fat‐to‐body weight ratio*, effects of diabetes: *F*_(1, 28)_ = 1742.2, *p* < 0.0001, effects of miR‐200a‐3p: *F*_(1, 28)_ = 1.51, *p* = 0.2293, interaction: *F*_(1, 28)_ = 0.35, *p* = 0.5570; *blood glucose levels*, effects of diabetes: *F*_(1, 28)_ = 35.0, *p* < 0.0001, effects of miR‐200a‐3p: *F*_(1, 28)_ = 2.85, *p* = 0.1023, interaction: *F*_(1, 28)_ = 2.74, *p* = 0.1091; *HbA*_*1C*_ *levels*, effects of diabetes: *F*_(1, 28)_ = 167.4, *p* < 0.0001, effects of miR‐200a‐3p: *F*_(1, 28)_ = 0.06, *p* = 0.8101, interaction: *F*_(1, 28)_ = 0.13, *p* = 0.7187).

### 3.6 Changes in Memory Function With Intraperitoneal Injection of miR‐200a‐3p Mimic

**FIGURE 5:**
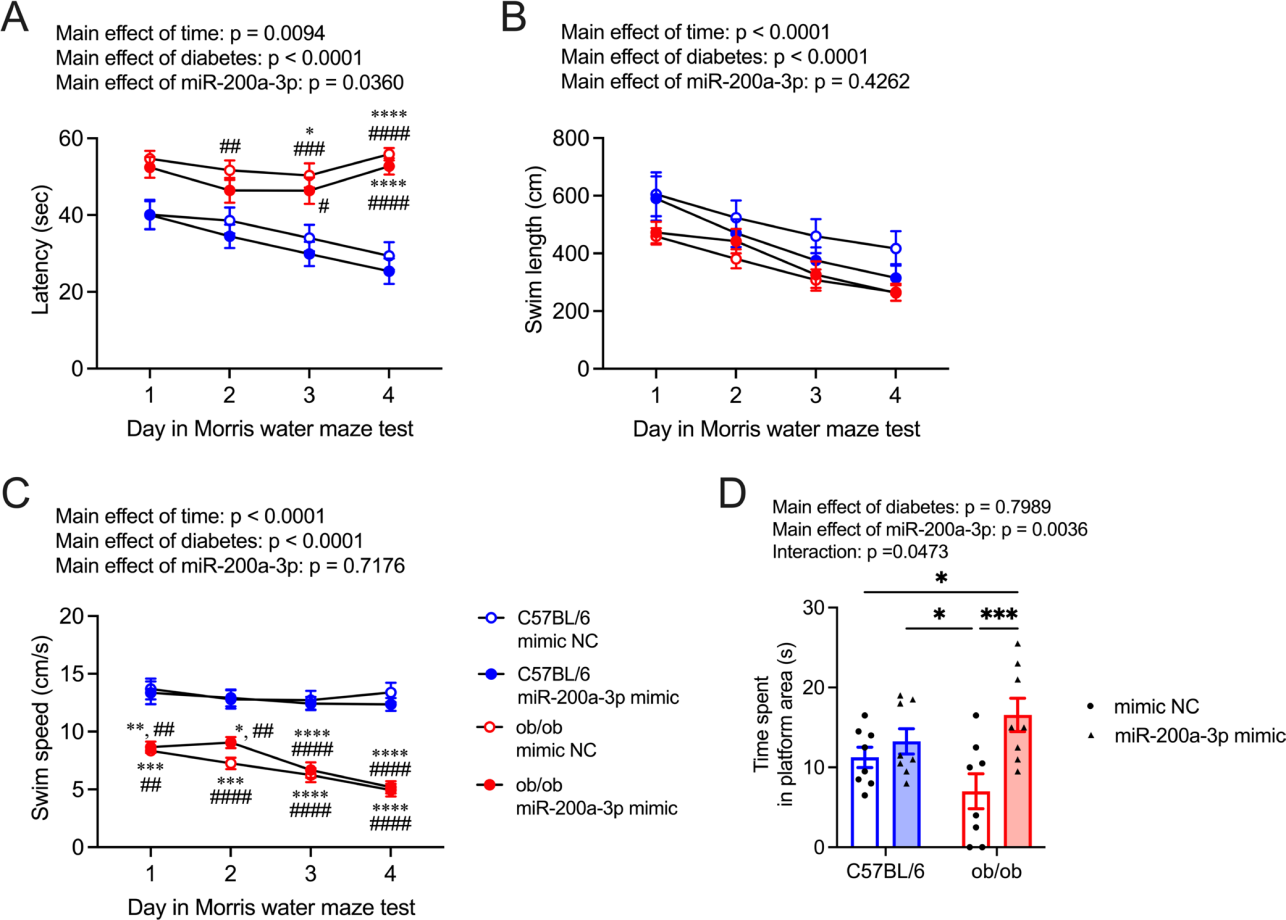
Effect of daily i.p. injection of mmu‐miR‐200a‐3p mimic on learning and memory function. Escape latency (A), swim length (B), and speed (C) during the learning session in mice (mean with 95% confidence intervals). Blue circles: C57BL/6 mice, red circles: Ob/ob mice, mimic NC: Mice treated with miRNA mimic negative control, miR‐200a‐3p mimic, mice treated mmu‐miR‐200a‐3p mimic. *p < 0.05, **p < 0.01, ***p < 0.001, ****p < 0.0001 versus C57BL/6 mimic NC, ^#^ *p* < 0.05, ^##^ *p* < 0.01, ^###^ *p* < 0.001, ^####^ *p* < 0.0001 versus C57BL/6 miR‐200a‐3p mimic. (D) Effect of exercise on the probe trial, showing the time spent in the platform area. Blue bars: C57BL/6 mice, red bars: Ob/ob mice, circles: Mice treated with miRNA mimic negative control, and triangles: Mice treated mmu‐miR‐200a‐3p mimic. *p < 0.05, ***p < 0.001. Data are expressed as mean with 95% confidence intervals, *n* = 8 mice for each group.

Ob/ob mice treated with miRNA mimic NC exhibited notably prolonged escape latency compared to both C57BL/6 mice treated with miRNA mimic NC and miR‐200a‐3p mimic; conversely, ob/ob mice treated with miR‐200a‐3p mimic did not exhibit this prolonged escape latency on third day of the learning session (Figure 5A; effects of day: *F*_(3, 372)_ = 3.88, effects of diabetes: *F*_(1, 124)_ = 118.5, effects of miR‐200a‐3p: *F*_(1, 124)_ = 4.50, interaction [day × diabetes]: *F*_(3, 372)_ = 4.72, *p* = 0.0030, interaction [day × miR‐200a‐3p]: *F*_(3, 372)_ = 0.25, *p* = 0.8582, interaction [diabetes × miR‐200a‐3p]: *F*_(1, 124)_ = 0.03, *p* = 0.8620, interaction [day × diabetes × miR‐200a‐3p]: *F*_(3, 372)_ = 0.05, *p* = 0.9862). Swim distance and swimming speed were influenced by time and T2DM, yet treatment with miR‐200a‐3p mimic did not impact these parameters (Figure 5B,C; *swim distance*, effects of day: *F*_(3, 372)_ = 15.42, effects of diabetes: *F*_(1, 124)_ = 16.76, effects of miR‐200a‐3p: *F*_(1, 124)_ = 0.64, interaction [day × diabetes]: *F*_(3, 372)_ = 0.16, *p* = 0.9225, interaction [day × miR‐200a‐3p]: *F*_(3, 372)_ = 0.30, *p* = 0.8228, interaction [diabetes × miR‐200a‐3p]: *F*_(1, 124)_ = 2.81, *p* = 0.0962, interaction [day × diabetes × miR‐200a‐3p]: *F*_(3, 372)_ = 0.17, *p* = 0.9195; *swimming speed*, effects of day: *F*_(3, 372)_ = 10.31, effects of diabetes: *F*_(1, 124)_ = 175.8, effects of miR‐200a‐3p: *F*_(1, 124)_ = 0.13, interaction [day × diabetes]: *F*_(3, 372)_ = 5.88, *p* = 0.0006, interaction [day × miR‐200a‐3p]: *F*_(3, 372)_ = 0.99, *p* = 0.3993, interaction [diabetes × miR‐200a‐3p]: *F*_(1, 124)_ = 1.47, *p* = 0.2272, interaction [day × diabetes × miR‐200a‐3p]: *F*_(3, 372)_ = 0.17, *p* = 0.9144). Furthermore, akin to light‐intensity exercise intervention, the daily intraperitoneal injection of miR‐200a‐3p mimic increased the times spent in the platform area during the probe test for ob/ob mice (Figure 5D). Conversely, ob/ob mice treated with miRNA mimic NC exhibited significantly shorter times spent in the platform area compared to C57BL/6 mice treated with miR‐200a‐3p mimic (Figure 5D; effects of diabetes: *F*_(1, 28)_ = 0.07, effects of miR‐200a‐3p: *F*_(1, 28)_ = 10.07, interaction: *F*_(1, 28)_ = 4.31).

### 3.7 Changes in miR‐200a‐3p Levels in the Plasma and the Hippocampus With Intraperitoneal Injection of miR‐200a‐3p Mimic

**FIGURE 6:**
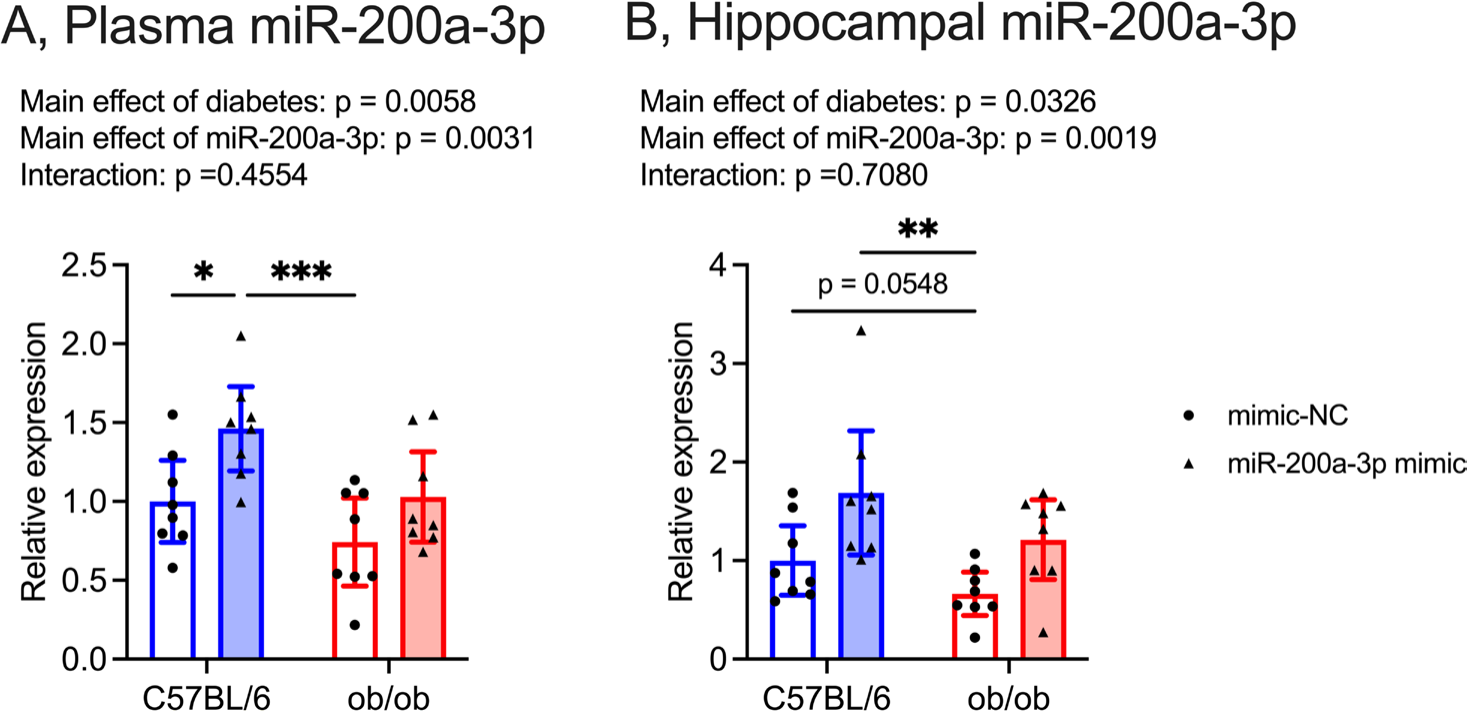
Effect of daily i.p. injection of mmu‐miR‐200a‐3p mimic on miR‐200a‐3p levels in the plasma (A) and the hippocampus (B) C57BL/6 mimic NC group was normalized as 1.0, mimic NC: Mice treated with miRNA mimic negative control, miR‐200a‐3p mimic, mice treated with mmu‐miR‐200a‐3p mimic. Data are expressed as mean with 95% confidence intervals, *n* = 8 mice for each group. *p < 0.05, **p < 0.01, ***p < 0.001.

Both plasma and hippocampal miR‐200a‐3p levels were significantly downregulated by T2DM and upregulated by miR‐200a‐3p mimic treatment (Figure 6A,B; *plasma miR‐200a‐3p*, effects of diabetes: *F*_(1, 28)_ = 8.92, effects of miR‐200a‐3p: *F*_(1, 28)_ = 10.47, interaction: *F*_(1, 28)_ = 0.57; *hippocampal miR‐200a‐3p*, effects of diabetes: *F*_(1, 28)_ = 5.06, effects of miR‐200a‐3p: *F*_(1, 28)_ = 11.72, interaction: *F*_(1, 28)_ = 0.14). In hippocampal miR‐200a‐3p levels, there was a trend towards decreased levels in ob/ob mice treated with miRNA mimic NC compared to C57BL/6 mice treated with miRNA mimic NC, but this trend was not evident in ob/ob mice treated with miR‐200a‐3p mimic (Figure 6B).

### 3.8 Changes in mRNA Levels in the Hippocampus With Intraperitoneal Injection of miR‐200a‐3p Mimic

**FIGURE 7:**
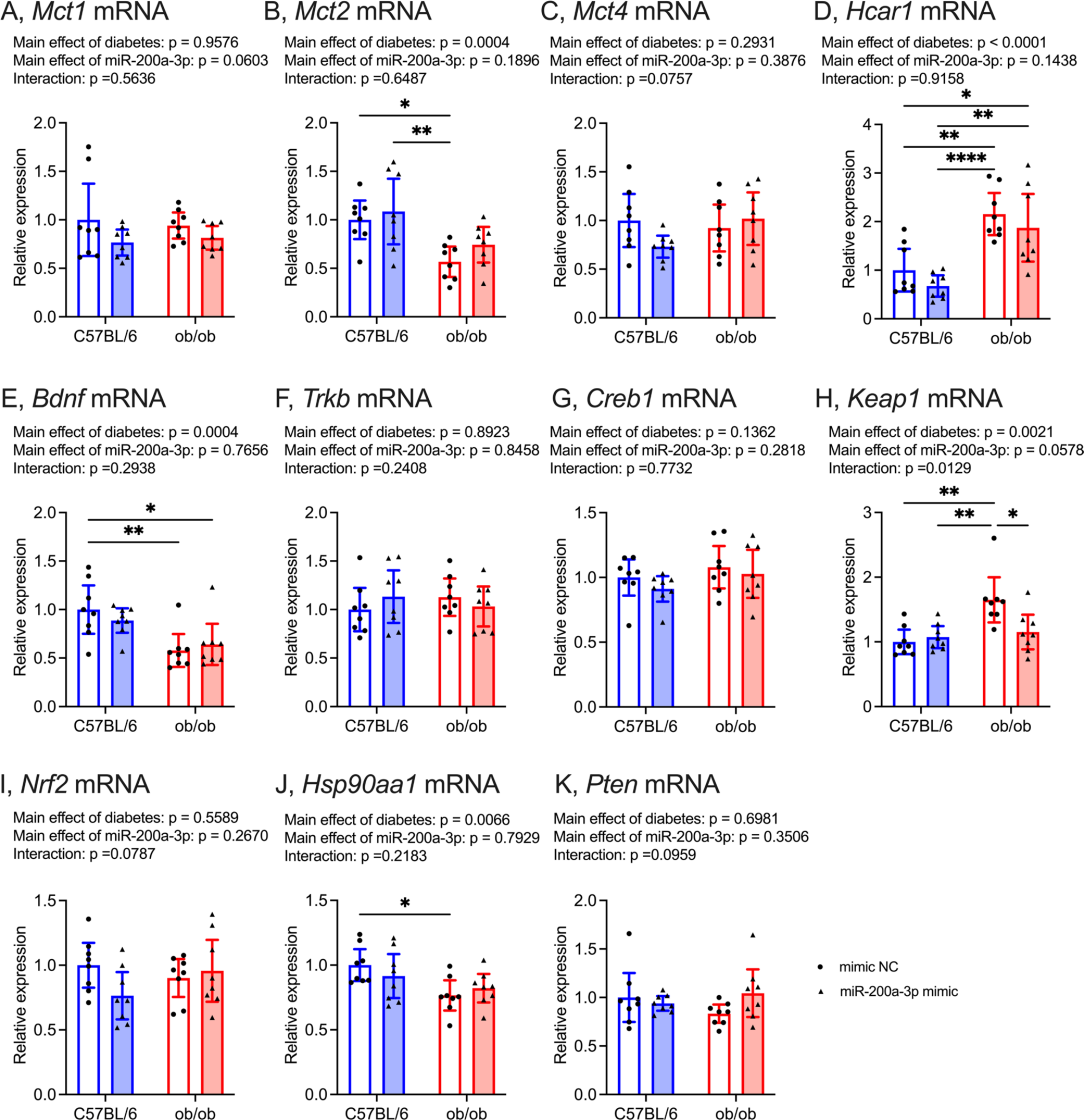
Effect of daily i.p. injection of mmu‐miR‐200a‐3p mimic on mRNA levels of *Mct1* (A), *Mct2* (B), *Mct4* (C), *Hcar1* (D), *Bdnf* (E), *Trkb* (F), *Creb1* (G), *Keap1* (H), *Nrf2* (I), *Hsp90aa1* (J), *Pten* (K) in the hippocampus. C57BL/6 mimic NC group was normalized as 1.0, mimic NC: Mice treated with miRNA mimic negative control, miR‐200a‐3p mimic, mice treated mmu‐miR‐200a‐3p mimic. Data are expressed as mean with 95% confidence intervals, *n* = 8 mice for each group. *p < 0.05, **p < 0.01, ****p < 0.0001.

Hippocampal mRNA levels of *Mct1* and *Mct4* remained unaffected by T2DM or miR‐200a‐3p mimic treatment (Figure 7A,C; *Mct1*, effects of diabetes: *F*_(1, 28)_ = 0.003, effects of miR‐200a‐3p: *F*_(1, 28)_ = 3.83, interaction: *F*_(1, 28)_ = 0.34; *Mct4*, effects of diabetes: *F*_(1, 28)_ = 1.15, effects of miR‐200a‐3p: *F*_(1, 28)_ = 0.77, interaction: *F*_(1, 28)_ = 3.40). However, the daily intraperitoneal injection of miR‐200a‐3p mimic notably improved *Mct2* mRNA levels in the hippocampus of ob/ob mice (Figure 7B; effects of diabetes: *F*_(1, 28)_ = 15.8, effects of miR‐200a‐3p: *F*_(1, 28)_ = 1.81, interaction: *F*_(1, 28)_ = 0.21). Furthermore, only ob/ob mice treated with miRNA mimic NC exhibited downregulated levels of *Mct2* mRNA compared to C57BL/6 mice (Figure 7B). Hippocampal *Hcar1* mRNA levels were significantly higher in T2DM mice than in control mice (Figure 7D; effects of diabetes: *F*_(1, 28)_ = 33.9, effects of miR‐200a‐3p: *F*_(1, 28)_ = 2.26, interaction: *F*_(1, 28)_ = 0.01). In contrast, both miRNA mimic NC‐ and miR‐200a‐3p mimic‐treated ob/ob mice exhibited significantly lower *Bdnf* mRNA levels in the hippocampus compared to C57BL/6 mice treated with miRNA mimic NC (Figure 7E; effects of diabetes: *F*_(1, 28)_ = 16.5, effects of miR‐200a‐3p: *F*_(1, 28)_ = 0.09, interaction: *F*_(1, 28)_ = 1.15). Hippocampal *Trkb* and *Creb1* mRNA levels remained unchanged by T2DM or miR‐200a‐3p mimic treatment (Figure 7F,G; *Trkb*, effects of diabetes: *F*_(1, 28)_ = 0.02, effects of miR‐200a‐3p: *F*_(1, 28)_ = 0.04, interaction: *F*_(1, 28)_ = 1.44; *Creb1*, effects of diabetes: *F*_(1, 28)_ = 2.35, effects of miR‐200a‐3p: *F*_(1, 28)_ = 1.20, interaction: *F*_(1, 28)_ = 0.09). Ob/ob mice treated with miRNA mimic NC solely exhibited upregulated levels of *Keap1* mRNA compared to all other groups (Figure 7H; effects of diabetes: *F*_(1, 28)_ = 11.5, effects of miR‐200a‐3p: *F*_(1, 28)_ = 3.91, interaction: *F*_(1, 28)_ = 7.05), and downregulated levels of *Hsp90aa1* mRNA compared to C57BL/6 mice treated with miRNA mimic NC (Figure 7J; effects of diabetes: *F*_(1, 28)_ = 8.61, effects of miR‐200a‐3p: *F*_(1, 28)_ = 0.07, interaction: *F*_(1, 28)_ = 1.59). Hippocampal *Nrf2* and *Pten* mRNA levels remained unaltered by T2DM or miR‐200a‐3p mimic treatment (Figure 7I,K; *Nrf2*, effects of diabetes: *F*_(1, 28)_ = 0.35, effects of miR‐200a‐3p: *F*_(1, 28)_ = 1.28, interaction: *F*_(1, 28)_ = 3.33; *Pten*, effects of diabetes: *F*_(1, 28)_ = 0.15, effects of miR‐200a‐3p: *F*_(1, 28)_ = 0.90, interaction: *F*_(1, 28)_ = 2.97).

## 4 Discussion

In the current study, we investigated the involvement of muscle‐derived exosomal miR‐200a‐3p in the improvement of spatial memory dysfunction in T2DM mice undergoing light‐intensity exercise intervention. Our findings indicated that light‐intensity exercise intervention restores gastrocnemius muscle‐derived exosomal miR‐200a‐3p levels, accompanied by improvement in spatial memory function and hippocampal *Mct2* mRNA levels in ob/ob mice. Additionally, the exercise intervention downregulated *Keap1* mRNA levels and upregulated *Hsp90aa1* mRNA levels in the hippocampus of ob/ob mice. Furthermore, the daily intraperitoneal injection of miR‐200a‐3p mimic in ob/ob mice not only alleviated spatial memory dysfunction but also mimicked the effects on the hippocampal RNA profiles observed with light‐intensity exercise intervention.

Consistent with previous reports [10, 11], the current study reaffirms the improvement of spatial memory dysfunction and hippocampal *Mct2* mRNA levels in ob/ob mice with light‐intensity exercise intervention (Figures 1 and 2B). The mRNA levels of *Mct1* and *Mct4* in the hippocampus, responsible for releasing lactate into the extracellular fluid from astrocytes, remained unaffected by both T2DM and the current light‐intensity exercise regimen (Figure 2A,C). MCT2 primarily facilitates the uptake of lactate into neurons [15, 47, 48], and the lactate transport through MCT2 is crucial for maintaining hippocampal function [16, 49, 50]; restoring hippocampal *Mct2* levels might partially contribute to light‐intensity exercise‐induced improvement of spatial memory dysfunction in T2DM mice. Interestingly, mRNA levels of *Hcar1*, a specific receptor for lactate, were upregulated in ob/ob mice (Figure 2D). HCAR1, also known as GPR81, could play roles in enhancing neuroplasticity and in exercise‐induced hippocampal adaptations [51, 52, 53]. Despite ob/ob mice exhibiting hippocampus‐based spatial memory dysfunction (Figure 1), hippocampal *Hcar1* mRNA levels of ob/ob mice were higher than those of control mice; notably, hippocampal *Hcar1* mRNA levels were unchanged with light‐intensity exercise intervention (Figure 2D). While further investigations are warranted to explore the sensitivity of hippocampal HCAR1 in T2DM, our findings suggest the importance of MCT2 in the light‐intensity exercise‐induced improvement of spatial memory dysfunction in T2DM.

Aligning with the improvement of memory dysfunction and hippocampal *Mct2* mRNA levels (Figure 2B), there were improvements in gastrocnemius muscle‐derived exosomal miR‐200a‐3p, plasma exosomal miR‐200a‐3p, and hippocampal miR‐200a‐3p with light‐intensity exercise intervention in ob/ob mice (Figure 3A–C). The current study, in accordance with a previous report [11], affirms the enhancement of miR‐200a‐3p levels in the hippocampus of ob/ob mice with the light‐intensity exercise regimen. Furthermore, the current results unveiled a significant positive relationship between exosomal miR‐200a‐3p secreted from the gastrocnemius muscle and those in plasma (Figure 3D). While exosomal miR‐200a‐3p levels in plasma exhibited a nonsignificant correlation with hippocampal miR‐200a‐3p levels (Figure 3E), the daily intraperitoneal injection of mmu‐miR‐200a‐3p mimic improved memory dysfunction, as well as plasma and hippocampal miR‐200a‐3p levels, and hippocampal *Mct2* mRNA levels in ob/ob mice (Figures 5, 6 and 7B). These findings suggest that the transport of peripheral miR‐200a‐3p into the hippocampus may play a role in maintaining hippocampal spatial memory function in T2DM. Although the precise origin of plasma exosomal miR‐200a‐3p, particularly whether it derives from the gastrocnemius muscle, remains unclear, our results indicate a potential contribution of peripheral miR‐200a‐3p to the beneficial effects of light‐intensity exercise in T2DM. Future studies should address this issue by labeling plasma exosomal miRNA with 5‐ethynyl uridine, a uridine analog, to determine its muscle‐derived origin [54].

Hippocampal *Keap1* mRNA levels were upregulated in T2DM and showed a tendency to decrease with light‐intensity exercise intervention (Figure 4A). Additionally, hippocampal *Pten* mRNA levels were significantly reduced with light‐intensity exercise intervention (Figure 4D). These results indicate that the light‐intensity exercise regimen in ob/ob mice led to improvements in hippocampal *Keap1* and *Pten* mRNA levels. Although both *Keap1* and *Pten* mRNA expressions are regulated by miR‐200a‐3p [31, 32], the current study has exhibited that hippocampal miR‐200a‐3p expressions in ob/ob mice exhibited a trend of negative correlation with *Keap1* mRNA levels but not with *Pten* mRNA levels (Figure 4F,G). Furthermore, both plasma and hippocampal miR‐200a‐3p levels were significantly reduced by T2DM and increased by miR‐200a‐3p mimic treatment (Figure 6A,B). The mmu‐miR‐200a‐3p mimic treatment decreased hippocampal *Keap1* mRNA levels but did not affect *Pten* mRNA levels in ob/ob mice (Figure 7H,K). These results suggest that the increase in hippocampal miR‐200a‐3p levels induced by light‐intensity exercise may contribute to the improvement of hippocampal *Keap1* mRNA levels in T2DM. KEAP1 inhibits the expression of NRF2 and HSP90 [33, 34]. The current light‐intensity exercise intervention improved *Hsp90aa1* mRNA levels, but not *Nrf2* mRNA levels, in the hippocampus of ob/ob mice (Figure 4B,C). HSP90 could contribute to enhancing hippocampal neuroplasticity [55, 56]. Patients with AD exhibit lower levels of HSP90 in their hippocampus compared to subjects without AD [57], and HSP90 may be related to the association between T2DM and AD [58]. The current treatment of mmu‐miR‐200a‐3p mimic led to changes in hippocampal *Hsp90aa1* mRNA levels in ob/ob mice, similar to the effects of light‐intensity exercise intervention (Figure 7I). Therefore, we might propose that KEAP1/HSP90 signaling, regulated by miR‐200a‐3p, plays a role in alleviating hippocampal dysregulation in T2DM. Although the profiles of HSPs relate to the changes in the expression of MCTs [40, 41], the direct relationship between HSP90 and MCT2 is unclear. Further mechanism‐focused studies, including ex vivo investigations and genetic modulation approaches, are necessary to elucidate this connection.

Consistent with previous reports [59, 60], hippocampal *Bdnf* mRNA levels were downregulated in ob/ob mice (Figures 2E and 7E). BDNF expression in the brain is known to be regulated by PTEN [39]; however, the alterations of hippocampal *Pten* mRNA associated with T2DM or exercise were inconsistent with the alterations of hippocampal *Bdnf* mRNA in the current study (Figures 2E, 4E, and 7E,K). Thus, the downregulated expression of BDNF in T2DM might not be directly linked to PTEN. BDNF modulates MCT2 expressions [42, 43], but hippocampal *Bdnf* mRNA levels and its downstream (*Trkb* and *Creb1* mRNA) in ob/ob mice remained unchanged with the current light‐intensity exercise (Figure 2E–G) or treatment of mmu‐miR‐200a‐3p mimic (Figure 7E–G), implying that the light‐intensity exercise restored spatial memory function and hippocampal *Mct2* levels in T2DM mice regardless of BDNF.

As shown in previous studies [11, 61, 62], elevated blood glucose and HbA_1C_ levels in ob/ob mice were improved with light‐intensity exercise (Table S2); however, the daily intraperitoneal injection of mmu‐miR‐200a‐3p mimic did not alter these biochemical parameters in ob/ob mice (Table S3). Therefore, it appears that miR‐200a‐3p may not have a direct impact on improving glycometabolism in peripheral organs. Instead, miR‐200a‐3p might represent a specific therapeutic strategy for targeting the hippocampus‐based spatial memory function in T2DM.

The current study has some limitations. Firstly, there is a need for further investigation into the specific mechanisms and interactions underlying how the uptake of lactate through hippocampal neuronal MCT2 is linked to memory dysfunction in T2DM. Future studies should explore lactate dynamics via MCTs using in vivo ^13^C NMR methods [63]. Secondly, our study focused solely on the hippocampal lactate transporter, neglecting the measurement of other molecular factors implicated in T2DM‐induced memory dysfunction, including angiogenesis, inflammation, oxidative stress, and insulin resistance [6, 64, 65]. For instance, a previous study has demonstrated a link between hippocampal insulin resistance and the downregulation of neuroplasticity and cognitive function [66]. Given that drugs enhancing insulin sensitivity and intranasal insulin injection have been shown to improve hippocampus‐based memory function in T2DM [67, 68, 69], hippocampal insulin resistance would be an important target to treat cognitive dysfunction associated with T2DM. Therefore, further studies are warranted to investigate the impact of the current interventions on insulin action in the hippocampus. Thirdly, the current study assessed hippocampal spatial memory function solely through the Morris water maze test in ob/ob mice, leaving the effects of the interventions on other type 2 diabetes animal models, such as high‐fat diet‐fed mice, unexplored. Additionally, the impact on other forms of hippocampus‐dependent cognitive functions, including novel object recognition and working memory, remains to be clarified. Additionally, memory function and circulating biochemical parameters in mice were evaluated only after the interventions, with no assessments conducted before the interventions. Fourthly, our study exclusively examined the effects of light‐intensity exercise. To determine the most effective exercise therapy for hippocampal treatment, comparisons with other exercise regimens, such as high‐intensity interval training [37, 70], are necessary. Fifthly, our specific emphasis on the modulation of *Keap1* and *Pten* mRNA by miR‐200a‐3p may not capture the full spectrum of its effects, as some studies have reported adverse outcomes, such as the promotion of neuronal apoptosis by miR‐200a‐3p [71]. Thus, a more comprehensive exploration of these aspects is warranted in future investigations. Sixthly, our measurements were restricted to mRNA levels, and we lack information on protein levels in the hippocampus. Based solely on the current findings, it is impossible to completely reveal the specific pathway of the interventions' effects. Hence, further mechanism‐based studies, including genetic modulations and ex vivo investigations, are warranted. Finally, it is essential to assess whether the results observed in animal models can be applied to human conditions in future studies.

In conclusion, our current findings represent that light‐intensity exercise intervention in ob/ob mice effectively ameliorates spatial memory dysfunction and enhances hippocampal *Mct2* mRNA levels, accompanied by the improvement of gastrocnemius muscle‐derived exosomal miR‐200a‐3p levels. The observed downregulation of hippocampal *Keap1* mRNA and upregulation of *Hsp90aa1* mRNA in response to both light‐intensity exercise and miR‐200a‐3p mimic treatment in ob/ob mice may suggest a potential involvement of the KEAP1/HSP90 signaling pathway modulated by miR‐200a‐3p in improving hippocampal dysregulation. These findings might contribute to the development of novel therapeutic strategies aimed at enhancing hippocampal spatial memory function in T2DM.

## Author Contributions

**Takeru Shima:** conceptualization, methodology, resources, investigation, writing – original draft, writing – review and editing, funding acquisition; **Hayate Onishi:** investigation, writing – review and editing; **Chiho Terashima:** investigation, writing – review and editing.

## Conflicts of Interest

The authors declare no conflicts of interest.

## Supporting information


Tables S1–S3.

## Data Availability

The datasets in the current study are available from the corresponding author on reasonable request.
