# Supplementary material for: Muscle‐Derived miR‐200a‐3p Through Light‐Intensity Exercise May Contribute to Improve Memory Dysfunction in Type 2 Diabetic Mice
Source: FASEB J. 2025 Apr 10;39(7):e70531. doi: 10.1096/fj.202500336R (PMC11983088; doi:10.1096/fj.202500336R)
Supplement: Supplementary file 1 — Tables S1–S3. [file FSB2-39-e70531-s001.docx]

**Supplementary Tables**

Supplementary Table 1. ﻿Primer sequences

| gene | F-primer | R-primer |
| --- | --- | --- |
| *Mct1* | GATGAGGACCAGAAGGTTCG | GATTGGGTAGTTCGGCATTG |
| *Mct2* | TGACGAGTTTGTCCAGGAGA | TTGCTGCTCTCATTGAGGC |
| *Mct4* | GTGTCGCTGTAGCCAATCCC | GGCTGTTTTATCATCACGGGTT |
| *Hcar1* | AGAAGAAGGATGTGCGGATG | TCTTGAAGAGCACAGGCTCA |
| *Bdnf* | GATGAGGACCAGAAGGTTCG | GATTGGGTAGTTCGGCATTG |
| *Trkb* | TGACGAGTTTGTCCAGGAGA | TTGCTGCTCTCATTGAGGC |
| *Creb1* | TCAGCCGGGTACTACCATTC | TCTCTTGCTGCTTCCCTGTT |
| *Keap1* | CTGCCCAATTCATGGCTCACA | CTTAGGGTGGATGCCTTCGAT |
| *Nrf2* | GAGGTCACCACAACACGAAC | ATCTCATAAGGCCCCACCTC |
| *Hsp90aa1* | GACGCTCTGGATAAAATCCGTT | TGGGAATGAGATTGATGTGCAG |
| *Pten* | TGGCGGAACTTGCAATCCTCAGT | TCCCGTCGTGTGGGTCCTGA |
| *β-actin* | TATGCCAACACAGTGCTGTCTGG | TACTCCTGCTTGCTGATCCACAT |

Supplementary Table 2. ﻿The effects of light-intensity exercise on physiological and biochemical variables

|  | C57BL/6 | | |  | ob/ob | | |
| --- | --- | --- | --- | --- | --- | --- | --- |
|  | sedentary |  | exercised |  | sedentary |  | exercised |
| Body weight (g) | 26.53 [25.57-27.49] |  | 25.19 [24.38-26.00] |  | 52.19 [49.44-54.94]^****,####^ |  | 52.11 [50.67-53.55]^****,####^ |
| Fat weight（mg/g body weight） | 3.82 [3.19-4.44] |  | 3.11 [2.71-3.51] |  | 55.83 [52.22-59.44]^****,####^ |  | 52.62 [48.80-56.44]^****,####^ |
| Blood glucose (mg/dL) | 145.10 [130.67-159.53] |  | 134.67 [119.13-150.20] |  | 216.11 [157.60-274.63]^**,##^ |  | 178.22 [148.59-207.86] |
| HbA_1C_ (%) | 4.29 [4.19-4.39] |  | 4.27 [4.14-4.39] |  | 7.67 [7.07-8.27]^****,####^ |  | 6.60 [6.22-6.98]^****,####,+++^ |
| Values are mean [95% confidence intervals]. ** p < 0.01,**** p < 0.0001 vs C57BL/6-sedentary, ### p < 0.001, #### p < 0.0001 vs C57BL/6-exercised, +++ p < 0.001 vs ob/ob-sedentary. | | | | | | | |

Supplementary Table 3. The effects of intraperitoneal injection of miR-200a-3p mimic on physiological and biochemical variables

|  | C57BL/6 | | |  | ob/ob | | |
| --- | --- | --- | --- | --- | --- | --- | --- |
|  | mimic NC |  | miR-200a-3p mimic |  | mimic NC |  | miR-200a-3p mimic |
| Body weight (g) | 26.43 [25.96-26.89] |  | 25.90 [25.60-26.20] |  | 53.99 [49.47-58.51]^****,####^ |  | 53.38 [50.45-56.30]^****,####^ |
| Fat weight（mg/g body weight） | 3.08 [2.49-3.66] |  | 2.25 [1.80-2.70] |  | 58.24 [52.94-63.54]^****,####^ |  | 55.86 [52.81-58.92]^****,####^ |
| Blood glucose (mg/dL) | 124.25 [102.88-145.62] |  | 123.50 [109.18-137.82] |  | 290.38 [208.29-372.46]^****,####^ |  | 217.00 [158.94-275.06]^*,#^ |
| HbA_1C_ (%) | 4.28 [4.04-4.51] |  | 4.24 [4.07-4.41] |  | 8.16 [7.17-9.16]^****,####^ |  | 8.35 [7.32-9.38]^****,####^ |
| Values are mean [95% confidence intervals]. * p < 0.05, **** p < 0.0001 vs C57BL/6-mimic NC, # p < 0.05, #### p < 0.0001 vs C57BL/6-miR-200a-3p mimic. | | | | | | | |
